# Enhanced green view index

**DOI:** 10.1016/j.mex.2022.101824

**Published:** 2022-08-19

**Authors:** Harish Puppala, Jagannadha Pawan Tamvada, Byungmin Kim, Pranav R. T. Peddinti

**Affiliations:** a School of Engineering and Technology, BML Munjal University, Sidhrawali, 122413, Gurugram, Haryana, India; b Southampton Business School, University of Southampton, SO17 1BJ, UK; c Department of Urban and Environmental Engineering, Ulsan National Institute of Science and Technology (UNIST), UNIST-gil 50, Ulju-gun, Ulsan 44919, South Korea; d Department of Civil Engineering, Pandit Deendayal Energy University, 382007 Gujarat, India

**Keywords:** Enhaced green view index (EGVI), Green view index (GVI), Street level greenery, Normalized difference vegetation index (NDVI), Visible atmospherically resistant index (VARI)

## Abstract

Quantifying street-level greenery has been the subject of interest for researchers as it has several implications for community residents. Green View Index (GVI) is a widely used parameter to compute the greenery along the streets. However, it does not account for the health of the greenery. The new Enhanced Green View Index (EGVI) that we propose computes the amount of greenery along the streets along with the health of the greenery.

• *The new indicator computes street-level greenery;*

• *Considers the health of vegetation while calculating greenery; and*

• *Helps to study the impact of street-level greenery on community residents precisely.*

## Graphical abstract

**Figure:**
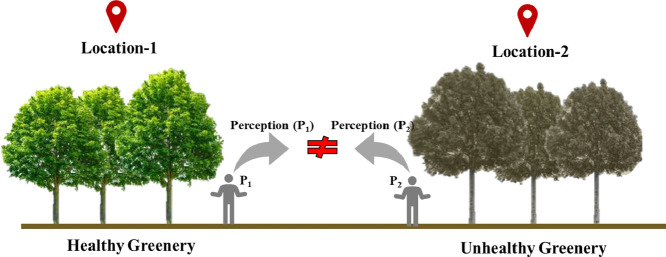

Specifications table

**Table:** 

Subject area:	Environmental Science
More specific subject area:	*Urban Sensing*
Name of your method:	*Enhanced Green View Index*
Name and reference of original method:	*GVI*
Resource availability:	*Not applicable*

## Background

Street-level greenery improves the aesthetics of the urban landscape and consequently has a substantial impact on the physical and mental health of the people [1]. It improves the walkability of the streets [2], community participation in doing physical exercise [3], and mental health of the community residents [4]. Street-level greenery helps in controlling the temperature of the environment [5], carbon sequestration [6], absorbing air pollutants, reducing noise levels, and controlling runoff due to precipitation [7]. Researchers have started quantifying street-level greenery to understand the relationship between greenery and its impacts. Green View Index (GVI), representing the fraction of green pixels in imagery captured along the streets, is one such indicator that is widely used in the literature [7]. In this regard, street-level urban sensing has gained new traction, with Google Street View (GSV) becoming a popular data repository providing 360^0^ views of the streets. GSV is considered a reliable audit tool that addresses the shortcomings of bias, resource, and time constraints of semi-structured interviews and questionnaire surveys used to determine the greenery on a qualitative scale [8]. Apart from GSV, Baidu Street View (BSV) and Tencent Street View (TSV) have also emerged as alternatives that primarily cater to the Chinese regions. Street-level data from GSV, TSV, and BSV have aided in computing the street-level greenery in terms of the Green View Index (GVI) [7,9,10], which represents the quantity of street-level greenery experienced by the users. As an alternative to the street view services, panoramic images captured using either a digital camera or a mobile are also be used to compute the GVI at a location.

**Fig. 1:**
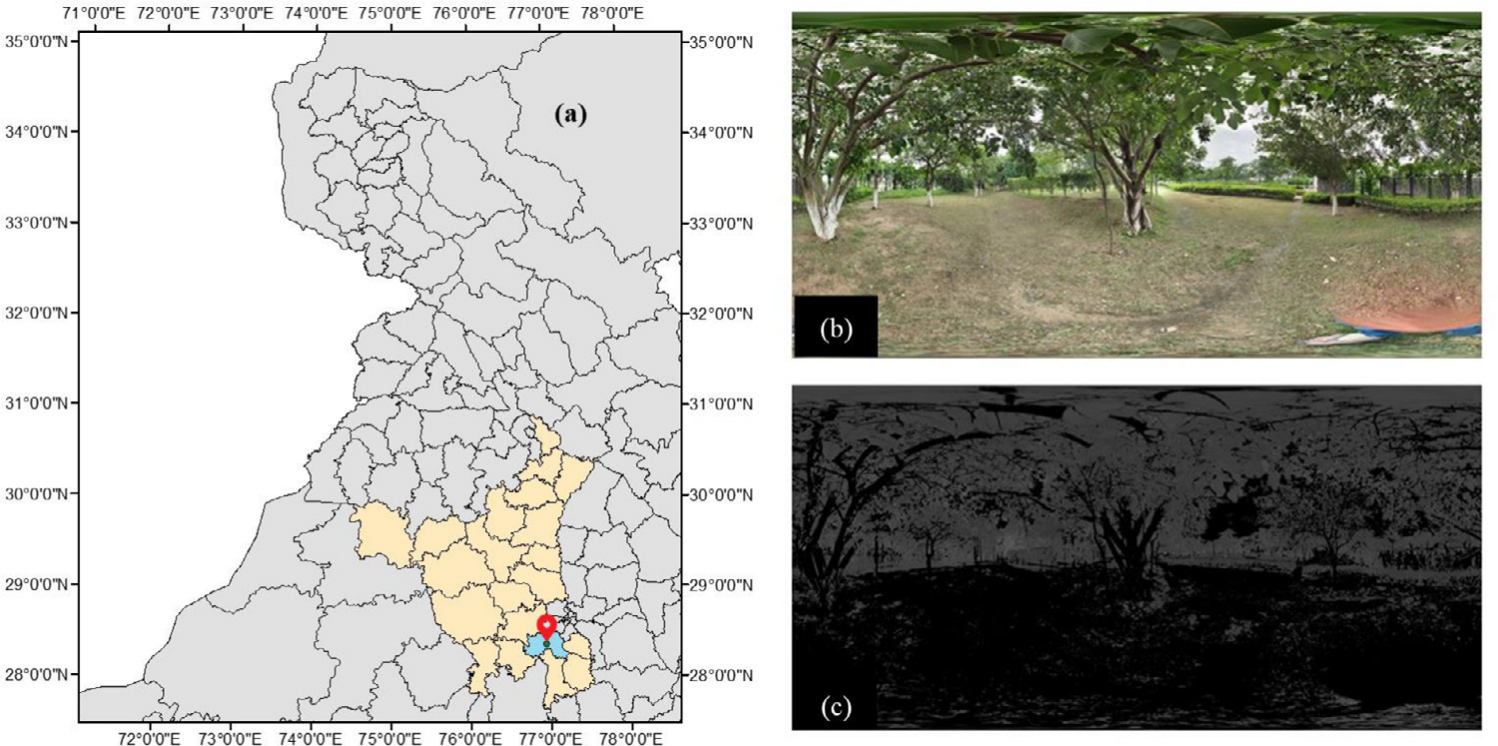
**(a)** Geographical location, (b) 360° view at a location, and (c) Classified image.

To illustrate the idea further, a 360° view at a location in Gurgaon district (Fig. 1(a)), India, is captured using a mobile camera (Fig. 1(b)). The image may be classified using a simple thresholding algorithm to classify each pixel into green and non-green pixels, as shown in Fig. 1(c). The count of each category is further used in computing the GVI at the location using Eq. 1, which is well known from the literature [7,11].

(1) $$GVI=\frac{Area\;of\;Greenery}{Total\;area}\times \;100$$

**Fig. 2:**
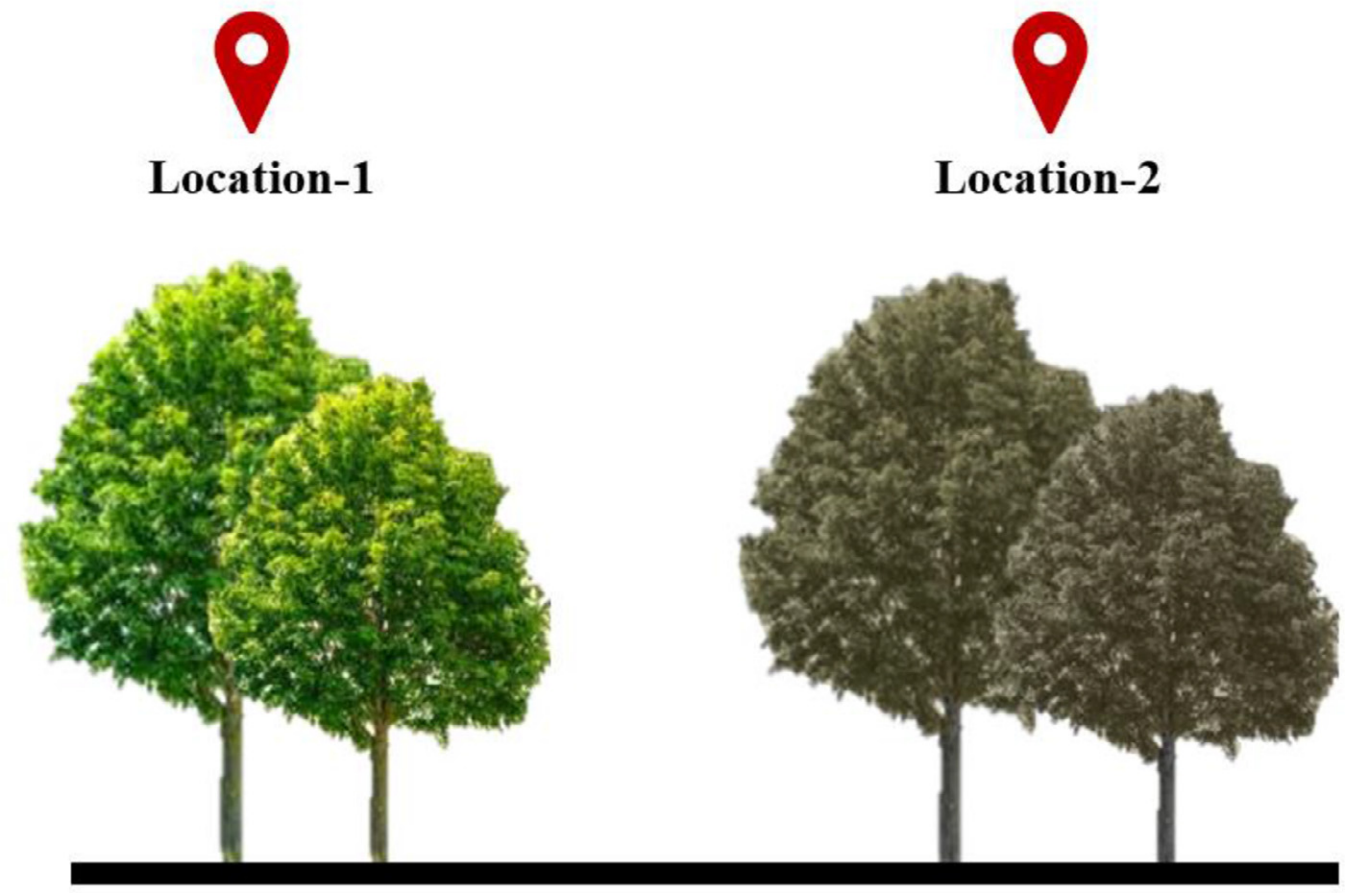
Photos of (a) healthy greenery and (b) unhealthy greenery.

Although GVI is an efficient indicator to capture the street-level greenery, an advanced model considering the effect of greenery health would be more advantageous. Greenery health influences the perception of community residents. To better understand this aspect, two different imageries with healthy and unhealthy greenery are created, as shown in Fig. 2a and b, respectively.

In both scenarios, the computed GVI results in the same score. However, when it comes to the perception of community residents, there will be a considerable difference in both scenarios, which can be apparent if the perception is collected on any chosen quantitative or qualitative scale. Owing to this limitation, this study envisages developing a new indicator that captures not only the quantity of greenery in a scene but also serves as a proxy measure of the impact that it can create on the perception of community residents.

## Method

A new indicator, Enhanced Green View Index (EGVI), is proposed in this study to capture fraction of greenery in a scene along with the health of the vegetation, which has a strong implication on human perception. Thus, quantifying EGVI at a location helps not only to quantify the greenery in a scene but also assists the planner in understanding the possible impact on human perception. EGVI can be computed using Eq. 2.

(2) $$EGVI\;=No.\;of\;green\;pixels\;\times \left(\frac{\frac{1}{n}\sum_{i=1}^{n}{\left(\frac{NIR-R}{NIR+R}\right)}_{i}\;}{Total\;number\;of\;pixels}\right)\times \;100$$

where, NIR, R refers to the Near Infrared and Red bands of a captured imagery, respectively. The numerator in the second term of Eq. 2 is well known as the Normalized Difference Vegetation Index (NDVI), which aids in accounting for the health of the vegetation. Thus, EGVI not only reflects the fraction of greenery in a scene but also the health of the vegetation. Since no readily available data sets provide the imagery of streets captured in the NIR band, an alternative approach to computing EGVI is shown in Eq .3.

(3) $$EGVI=No.\;of\;green\;pixels\;\times \left(\frac{\frac{1}{n}\sum_{i=1}^{n}{\left(\frac{G-R}{G+R-B}\right)}_{i}}{Total\;number\;of\;pixels}\right)\times \;100$$

where R, G, B refers to red, green, and blue bands of the captured imagery. The numerator in the second term of Eq. 3 is well known as the Visible Atmospherically Resistant Index (VARI), which also refers to the health of the vegetation. NDVI determines the absolute health of green plants, while the VARI measures how green each pixel is compared to other pixels.

**Fig. 3:**
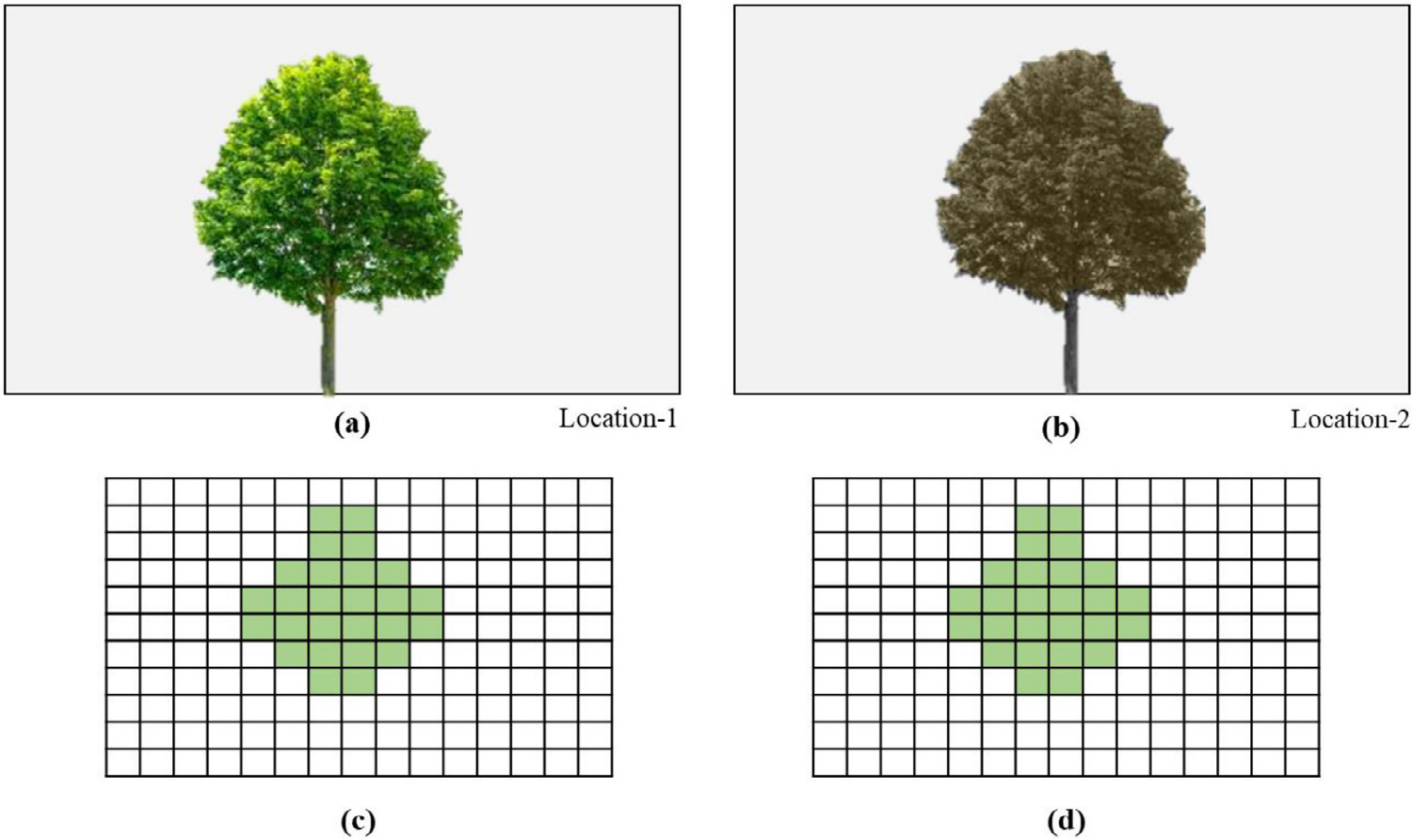
Synthesized image for demonstration: (a) health greenery; (b) unhealthy greenery; (c) terrestrial photograph of location-1; and (d) Terrestrial photograph of location-2.

To better understand the differences between the GVI and EGVI, a sample computation is shown in Fig. 3. Fig. 3(a) and (b) present the greenery at two different locations. Greenery in Fig. 3(a) is healthy compared to the greenery in Fig. 3(b). These images are synthesized to ensure that the dimension of the tree is similar in both of locations. Fig. 3(c) and (d) represents the classified image of location-1 and location-2, respectively. For a better understanding of the GVI and EGVI computations, a sample calculation is shown below.

The specifications of the captured images are as follows:

Total number of pixels in the terrestrial photograph captured at location-1 = 165 pixels
Total number of pixels in the terrestrial photograph captured at location-2 = 165 pixels
Total number of green pixels in the terrestrial photograph captured at location-1= 26 pixels
Total number of green pixels in the terrestrial photograph captured at location-2= 26 pixels
Assumed NDVI/VARI of greenery at location-1= 0.9 (considered a uniform value for the entire tree)
Assumed NDVI/VARI of greenery at location-2 = 0.4(considered a uniform value for the entire tree)

Considering the attributes above, the GVI and EGVI at location-1 and location-2 are computed as follows

At location-1 $GVI=\frac{26}{165}\times \;100=15.7$
At location-2 $GVI=\frac{26}{165}\times \;100=15.7$
At location-1 $EGVI\;=26\;\times (\frac{0.9\;}{165})\times \;100=14.13$
At location-2 $EGVI\;=26\;\times (\frac{0.4\;}{165})\times \;100=6.28$
Apart from this numerical example demonstrated using synthesized data, a pilot study is considered to exemplify the difference between GVI and EGVI.

### Pilot study

**Fig. 4:**
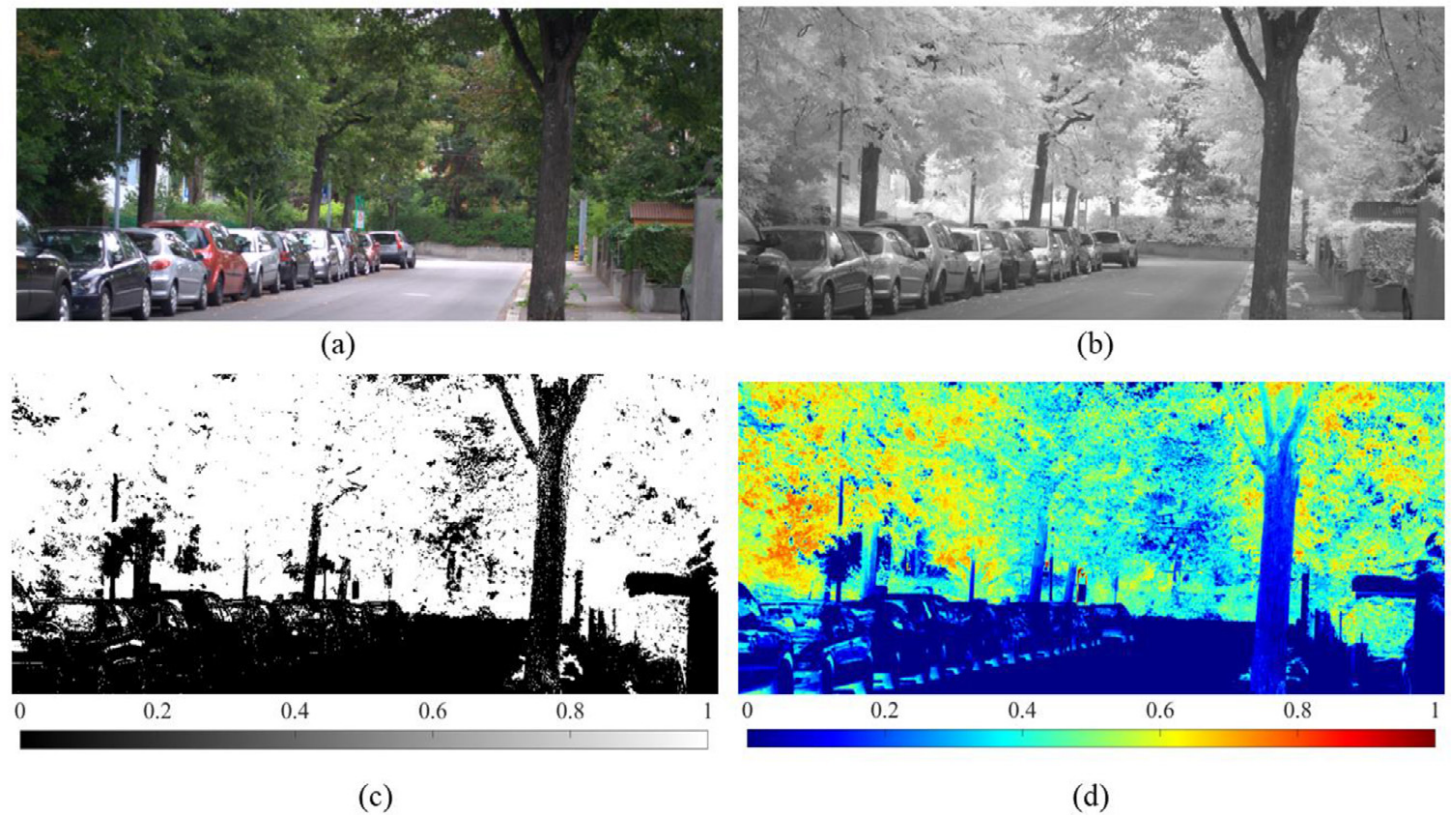
Terrestrial and processed imagery: (a) RGB imagery; (b) NIR Imagery; (c) Classified imagery; and (d) NDVI.

To demonstrate the proposed method, RGB and NIR images of a location are considered, as shown in Fig. 4(a) and (b), respectively**.** The RGB image is converted into an HSV scale, and considering the typical HSV range of green color, the image is classified using a simple thresholding algorithm to identify the green pixels. The classified image is shown in Fig. 4(c). Semantic segmentation and advanced machine learning techniques can also be used for image classification.

**Fig. 5:**
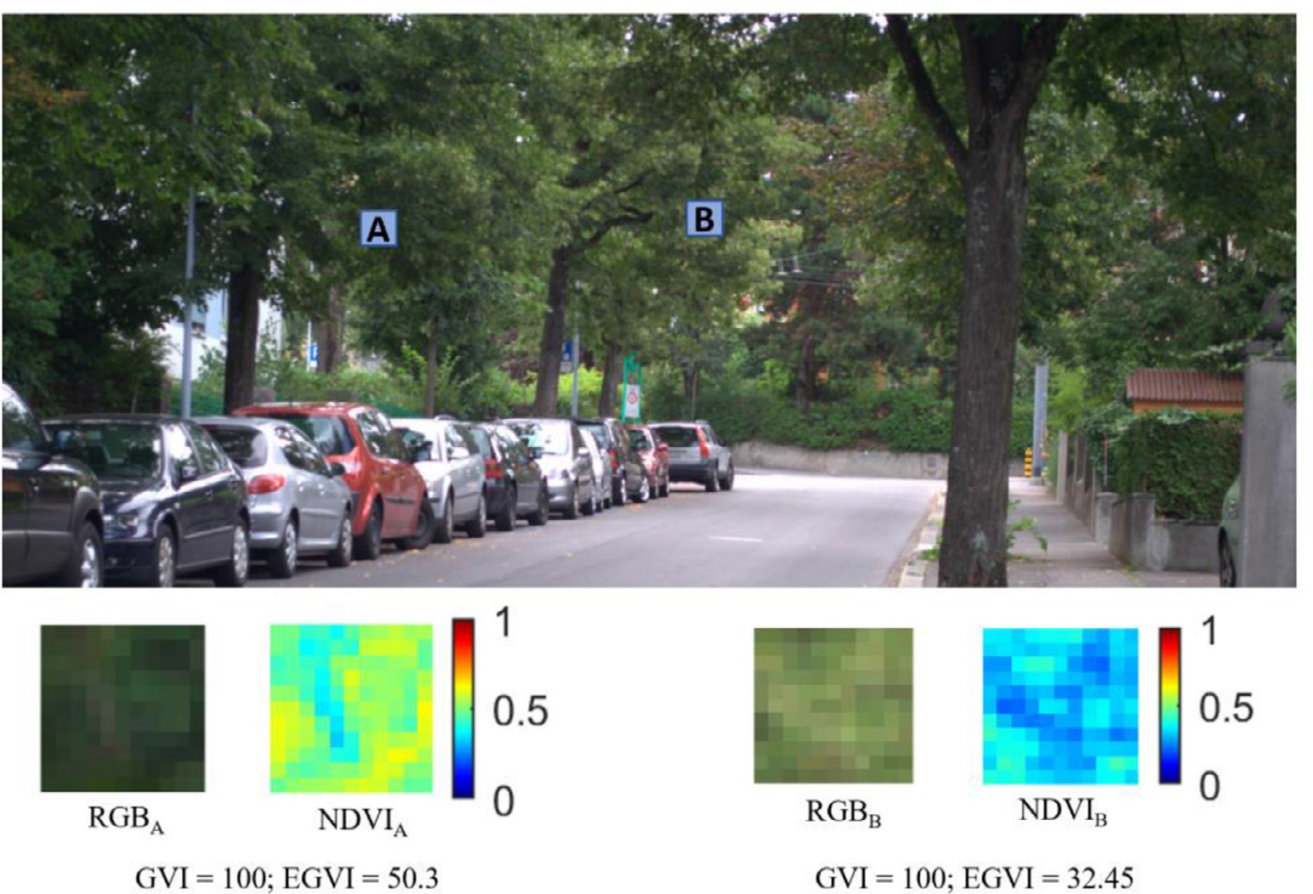
Variation of GVI and EGVI at two selected regions.

Later, red, green, and blue bands of the RGB image are exported as separate images. The created imagery of the red band and the NIR are used to determine the NDVI. Fig. 4(d) presents the spatial variation of NDVI within the considered scene, from which it is visually apparent that the NDVI of green pixels is not uniform. This infers that the health of vegetation throughout the scene is not the same, indicating that the considered scene contains both healthy and unhealthy vegetation. The subplots of Fig. 4 are used to compute GVI and EGVI using Eqs. 1 and 2, respectively. GVI and EGVI at the chosen location are 62.89 and 29.6, respectively. To explicitly monitor the difference between GVI and EGVI, two sub-regions (A and B), as shown in Fig. 5, are selected, and variation in respective GVI and EGVI is observed.

RGB_A_ and RGB_B_ in Fig. 5 show the vegetation in the selected sub-regions A and B, respectively. Since the regions chosen comprise only vegetation, the GVI representing the fraction of greenery is obtained as 100, indicating that the complete scene is green. Though the entire scenery within A and B is green, there is a difference in the intensity of green which is apparent from NDVI_A_ and NDVI_B_ in Fig. 5, implying that the health of greenery is different in both sub-regions. This difference in health could impact human perception towards greenery. EGVI, the proposed indicator, could capture this difference and distinguish A and B sub-regions. EGVI at A, where relatively healthy vegetation compared to B, is 50.3, while the EGVI at B is 32.45. This observation strengthens that EGVI could not only monitor the fraction of greenery but also account for the health.

As an alternative to NDVI used in the aforementioned computation, VARI can also be used to compute EGVI using Eq. 3 if there is no access to the NIR camera. However, it is to be noted that VARI does not reflect the true health of the vegetation. Therefore, the relative comparison of VARI-based EGVI requires calibration of all the captured images within a study area. Owing to this resource-intensive process, using Eq. 2 over Eq. 3 is recommended while computing EGVI.

From the findings of this study, it is evident that there is a substantial variation between GVI and EGVI, which can lead to significant differences in the perceptions of the users. Thus, drawing conclusions by considering the spatial variation of GVI may not be credible as the health of vegetation is not captured. The proposed index, i.e., EGVI, overcomes this limitation, providing a more comprehensive approach to understand street-level greenery and study its implications on human perception. This index can further be used for forestry health calculations by adding more attributes to vegetation cover data.

## Data availability

No data was used for the research described in the article.

## CRediT author statement

**Harish Puppala:** Conceptualization, Methodology, Writing- Original draft preparation. **Jagannadha Pawan Tamvada:** Conceptualization, Writing- Original draft preparation. **Byungmin Kim:** Conceptualization, Review and editing. **Pranav R T Peddinti:** Conceptualization, Writing- Review and editing.

## Declaration of Competing Interests

The authors declare that they have no known competing financial interests or personal relationships that could have appeared to influence the work reported in this paper.
